# IF YOU KNOW ONE CAREGIVER, YOU KNOW ONE CAREGIVER: UNDERSTANDING THE DIVERSITY OF US CAREGIVING EXPERIENCES

**DOI:** 10.1093/geroni/igad104.0794

**Published:** 2023-12-21

**Authors:** Rita Choula, Susan Reinhard, Selena Caldera, Ari Houser

**Affiliations:** AARP, Washington, District of Columbia, United States; AARP, Washington, District of Columbia, United States; AARP, Washington, District of Columbia, United States; AARP Public Policy Institute, Washington, District of Columbia, United States

## Abstract

For many years, the image of a family caregiver has been of a white woman in her late 40s providing care to her much older mother. The shifting demographics of the U.S. population have lead to a new understanding of the heterogeneity of family caregiving experiences and arrangements. Delays in having children and increasing longevity have led to an increase in caregivers sandwiched between care for someone under 18 at home and an older family member or friend and the share Gen Z and Millennial caregivers. The increasing racial and ethnic diversity and acceptance and visibility of persons identifying as LGBTQ has been reflected in the population of caregivers. Each of these communities of family caregivers have distinct care experiences and face unique challenges providing that essential care to family and friends with chronic, disabling, or serious health conditions. This presentation explores the diversity of the U.S. population of family caregivers and their unique care experiences detailed in the Valuing the Invaluable: 2023 Update. An overview of existing survey data on caregivers and quantitative and qualitative research into distinct caregiver experiences will provide attendees with a nuanced understanding of who family caregivers are and what they and their care recipients experience. In summary, the range of challenges these different caregivers face and positive experiences will be discussed.

